# A pilot study to assess bacterial and toxin reduction in patients with *Clostridium difficile* infection given fidaxomicin or vancomycin

**DOI:** 10.1186/s12941-016-0140-6

**Published:** 2016-04-12

**Authors:** Abrar K. Thabit, M. Jahangir Alam, Mohammed Khaleduzzaman, Kevin W. Garey, David P. Nicolau

**Affiliations:** Center for Anti-infective Research and Development, Hartford Hospital, 80 Seymour Street, Hartford, CT 06102 USA; Faculty of Pharmacy, King Abdulaziz University, Jeddah, Saudi Arabia; University of Houston College of Pharmacy, Houston, TX USA; Division of Infectious Diseases, Hartford Hospital, Hartford, CT USA

**Keywords:** *Clostridium difficile* toxins, Fidaxomicin, Vancomycin, Vegetative cells

## Abstract

**Background:**

To assess the effect of fidaxomicin and vancomycin on *Clostridium difficile* toxins and correlation with clinical and microbiologic outcomes.

**Methods:**

Hospitalized patients with *C. difficile* infection were randomly assigned a 10-day course of fidaxomicin or vancomycin. Stool samples collected at baseline (day 0), mid-therapy (days 3–5), end of therapy (days 10–13) and follow-up (days 19–38) were assessed for quantity of toxins A and B as well as spore and vegetative cells counts. Correlation of toxins concentrations with microbiologic and clinical findings were evaluated.

**Results:**

Among 34 patients 12 had detectable toxin concentrations at baseline seven were randomized to fidaxomicin and five to vancomycin. Overall both fidaxomicin and vancomycin resulted in drop of both toxins concentrations by midpoint of therapy. The drop in toxin A concentrations was maintained up to the follow-up period with fidaxomicin but not with vancomycin even in patients who developed recurrence. Patients who developed recurrence in the fidaxomicin group had lower concentrations of toxin B versus the recurrence patient of vancomycin group. Presence of vegetative cells and spores was significantly linked with high toxin A (*P* = 0.003 and <0.001 respectively) and toxin B (*P* = 0.007 and <0.001 respectively) concentrations across time points. Toxin B concentrations but not A significantly correlated with stool consistency (*P* < 0.001) and frequency (*P* = 0.05).

**Conclusions:**

Fidaxomicin was associated with sustained reduction of both toxins up to 30 days post therapy versus vancomycin. Multiple clinical or microbiologic observations were correlated with toxin A or B concentrations.

## Background

*Clostridium difficile* is a Gram-positive, anaerobic, spore-forming, organism that produces two toxins, A and B, which represent the major virulence factors of the organism [1]. *C. difficile* infection (CDI) results primarily from the effects of these toxins on the intestine causing fluid accumulation, epithelial inflammation, diarrhoea, pseudomembranous colitis, and death in severe cases [2–4]. The therapeutic goal for the management of CDI is to administer antimicrobials that are active against the pathogen, thereby reducing the bacterial burden and production of toxins A and B [5]. However, the decrease of bacterial burden and toxin quantity based on choice of therapy is poorly understood. A previously conducted in vitro study found that fidaxomicin but not vancomycin or metronidazole inhibited the production of *C. difficile* toxins A and B [6]. The objective of our current pilot investigation was to assess the in vivo effect of fidaxomicin and vancomycin on *C. difficile* toxin A and B quantity and assess for clinical correlations.

## Methods

### Patients and samples

From October 2012 through December 2014, patients admitted to Hartford Hospital, Hartford, CT, USA with CDI confirmed by PCR (GeneXpert^®^, Cepheid, Inc.; Sunnyvale, CA) were screened for an open-label, randomized study comparing the effects of fidaxomicin and vancomycin on microbiologic outcomes of CDI [7]. Less than 24 h of anti-CDI treatment was allowed prior to entry into the study. Patients were treated orally for 10 days with either fidaxomicin 200 mg every 12 h or vancomycin 125 mg every 6 h. Stool samples were collected at baseline (before therapy initiation, day 0), midpoint of therapy (days 3–5), end of therapy (days 10–13) and follow-up (days 19–38). The study protocol was approved by the Hartford Hospital Institutional Review Board, and all patients provided written, informed consent prior to entry into the study.

### Quantification of *C. difficile* toxins

*C. difficile* toxins A and B were quantified separately by *C. difficile* toxin A or B quanti kit (tgcBiomics GmbH, Germany) using sandwich enzyme-linked immunoassay (ELISA) [8]. Stool samples (50 μL if liquid/semiliquid or 50 mg if solid) were diluted at a ratio of 1:10 by dilution buffer (450 μL) provided with the kit. Samples were then homogenized with disposal pipetting suction and ejection or vortexing followed by centrifugation at 2500×*g* for 5 min. One hundred μL of clean supernatant was pipetted and poured into the microtiter plate well for toxin A or B analysis. Plates were read by Synergy HT microplate reader (BioTek Instruments, Inc., Winooski, VT) and measured optical density was analysed into respective concentration values by Gen5 software version1.09 (BioTek Instruments, Inc., Winooski, VT). The lower and upper limits of detection of the assay were 0.3 and 80 ng/mL, respectively, for both toxin A and B.

### Quantification of *C. difficile* bacterial counts

Quantification of *C. difficile* spores and vegetative cells was undertaken as previously described [9]. In brief, samples were cultured within 24 h of collection and colony forming units (CFU) were manually counted after incubation at 37 °C. Two aliquots of the sample were prepared; one was plated after treatment with ethanol in order to kill vegetative cells and keep spores, while the other was plated prior to ethanol treatment to keep both the spores and vegetative cells. Subtracting the number of spore CFUs in the ethanol-treated plate from the total number of CFUs in the untreated plate equalled the count of vegetative cells CFUs.

### Definitions

CDI clinical cure was defined as normalization of stool consistency and reduction of stool frequency to less than three unformed stools per day by day 10 of therapy. Clinical failure was defined as worsening of CDI with subsequent need for additional treatment beyond 10 days or a change in antibiotic therapy. Recurrence of CDI (defined as the presence of *C. difficile* along with clinical signs and symptoms of CDI) during the follow-up period was considered clinical failure for that time period. Asymptomatic colonization assessed during the follow-up period was defined as presence of *C. difficile* in stool without clinical symptoms of CDI. Stool consistency was visually determined for each collected sample using Bristol stool scale [10]. Stool frequency was determined based on the number of bowel movements over the last 24 h prior to sample collection.

### Analysis

To be included in the analysis, a stool sample with detectable toxin A or B at baseline was required. Differences between the fidaxomicin and vancomycin groups in terms of reduction in toxins concentrations from baseline compared to each subsequent sampling time were numerically described in the overall population of each treatment group, as well as after stratification for clinical cure and failure at end of therapy and follow-up. Spearman correlation (*r*_*s*_) was used to assess the correlation between vegetative cells to toxins concentrations, as well as to assess the correlation of the latter to stool consistency and frequency. A *P* value of 0.05 was considered statistically significant. All analyses were conducted using SigmaPlot version 12.5 (Systat Software, Inc., San Jose, CA, USA).

## Results

### Patients

**Fig. 1:**
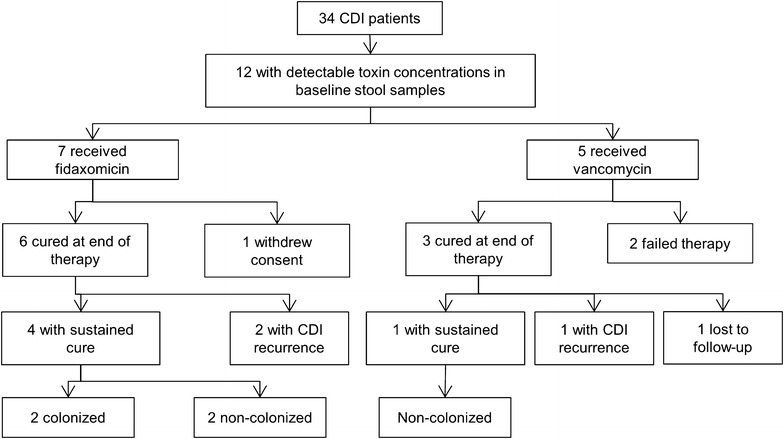
Flowchart of patients included in the study. *CDI* *Clostridium difficile* infection

Thirty four patients with CDI were enrolled during the study time period (Fig. 1). Of these 34 patients, 12 had detectable concentrations of toxin A or B in their baseline stool samples. Of these 12 patients, seven were among the fidaxomicin group and five in the vancomycin group. Patients given fidaxomicin or vancomycin were similar with regards to baseline characteristics (*P* > 0.3 for all comparisons) (Table 1).

**Table 1:** Baseline characteristics of patients infected with *Clostridium difficile* who had detectable toxins concentrations in their baseline stools

Demographic	Fidaxomicin	Vancomycin	*P* value
	N = 7	N = 5	
Age, years	68 ± 18	73 ± 12	0.87
Female, n (%)	3 (43 %)	3 (60 %)	1
Community-acquired CDI^a^, n (%)	4 (57 %)	2 (40 %)	1
BI/NAP1/027 strain, n (%)	5 (71 %)	2 (40 %)	1
Average unformed stool per day, n	6	6	1
Patients by bristol stool score, n (%)			0.4
7	5 (71 %)	3 (60 %)	
6	0	1 (20 %)	
≤5	2 (29 %)	1 (20 %)	
Baseline toxin A concentration (ng/mL)	0.31 [0.31–80]	3.99 [2.32–63.47]	0.53
Baseline toxin B concentration (ng/mL)	42.2 [23.62–80]	0.31 [0.31–80]	0.43
Baseline total CFU, log_10_ CFU/g stool	6.05 ± 2.2	6.8 ± 0.9	0.48
Baseline spores CFU, log_10_ CFU/g stool	5.44 ± 1.7	5.88 ± 1.24	0.75
Baseline vegetative cells CFU, log_10_ CFU/g stool	4.85 ± 2.85	6.57 ± 0.9	0.3
Previous therapy within 24 h, n (%)			0.66
Metronidazole	2 (29 %)	2 (40 %)	
Vancomycin	0	0	
Vancomycin + Metronidazole	3 (42 %)	3 (60 %)	
No therapy	2 (29 %)	0	

^a^CDI diagnosis in patients who had not been discharged from a healthcare facility in the previous 12 weeks [11]

Among the 7 patients given fidaxomicin, one withdrew consent prior to end of therapy while the remaining six experienced clinical cure. Of these six patients, two experienced CDI recurrence during the follow-up period. Of the remaining four patients given fidaxomicin, 2 were asymptomatically colonized with *C. difficile* during the follow-up period. Among the 5 patients given vancomycin, three experienced clinical cure. Of these 3 patients, one experienced CDI recurrence during the follow-up period. Of the remaining two patients, one was lost to follow-up and the other remained in remission and was non-colonized with *C. difficile.* Thus, in total 9 of 12 were clinically cured at end of therapy, 5 of 9 patients had a sustained clinical cure at follow-up while 3 of 9 experienced CDI recurrences.

### Change in stool *C. difficile* toxin A and B concentrations based on receipt of fidaxomicin or vancomycin

**Fig. 2:**
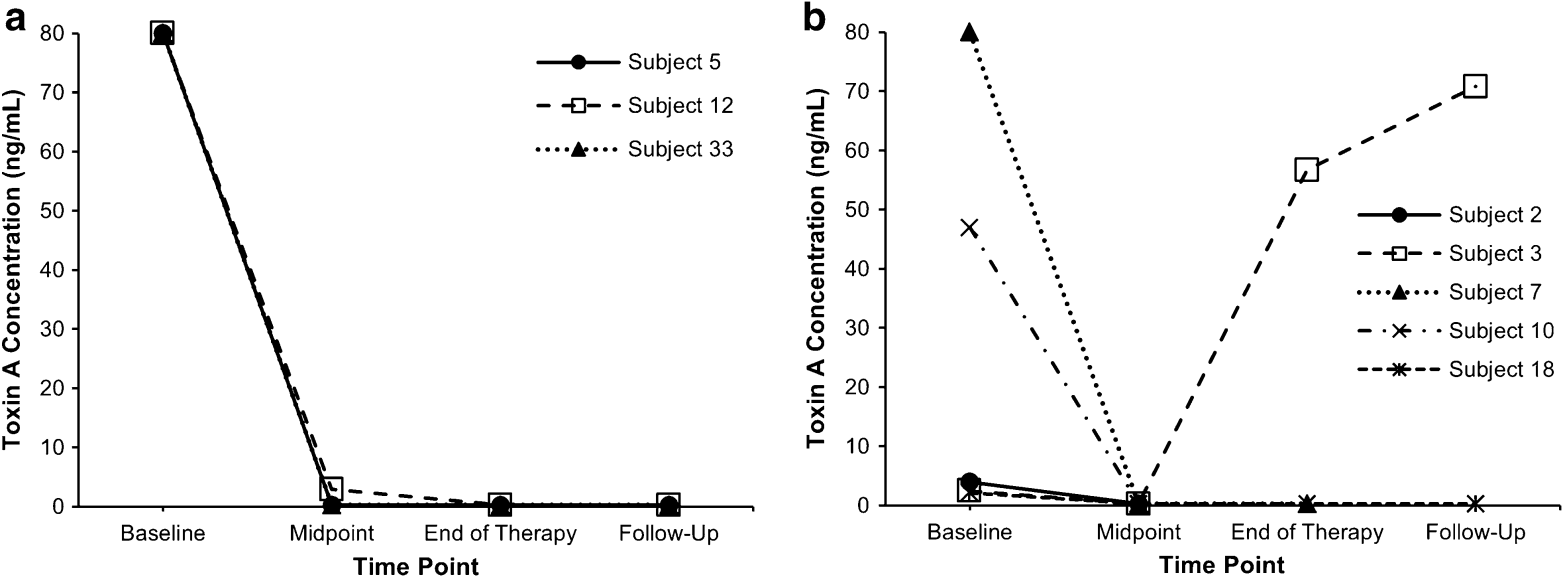
Toxin A concentrations (ng/mL) of individual patients in the fidaxomicin (**a**) and vancomycin (**b**) groups at each time point. *Only patients with detectable toxin B concentration at any point are presented. Subjects 5, 12, and 3 had CDI recurrence; whereas subject 33 had asymptomatic colonization

**Fig. 3:**
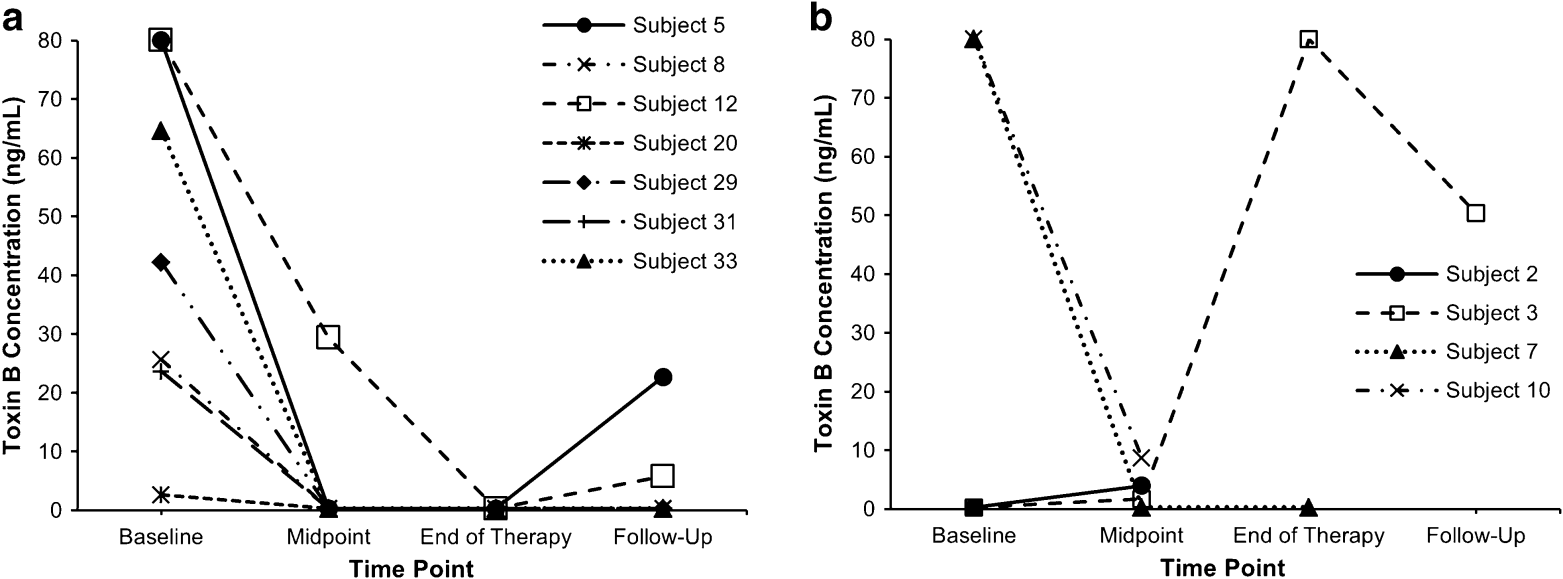
Toxin B concentrations (ng/mL) of individual patients in the fidaxomicin (**a**) and vancomycin (**b**) groups at each time point. *Only patients with detectable toxin B concentration at any point are presented. Subjects 5, 12, and 3 had CDI recurrence; whereas subject 33 had asymptomatic colonization

Baseline and subsequent concentrations of toxins A and B at each sampling time stratified by treatment with fidaxomicin or vancomycin are shown in Table 2. Figures 2 and 3 illustrate concentrations of toxins A and B throughout the duration of the study in individual patients. Proportion of samples with any detectable toxin in the whole patient population decreased from 100 % at baseline to 33 % at the mid-point evaluation, 11 % at the end of therapy, and 37 % at the follow-up evaluation.

**Table 2:** Faecal concentrations of *C. difficile* toxins A and B stratified by therapy with vancomycin or fidaxomicin

	Baseline	Mid-point	End-of-therapy	Follow-up
Toxin A				
Fidaxomicin	34.5 ± 39.4 (42.8 %); n = 3/7	0.7 ± 0.9 (14.3 %); n = 1/7	0.3 ± 0 (0 %); n = 0/6	0.3 ± 0 (0 %); n = 0/6
Vancomycin	27.1 ± 31.5 (100 %); n = 5/5	0.3 ± 0 (0 %); n = 0/5	19.1 ± 26.6 (33.3 %); n = 1/3	35.5 ± 35.2 (50 %); n = 1/2
Toxin B				
Fidaxomicin	45.5 ± 27.9 (100 %); n = 7/7	4.5 ± 10.2 (14.3 %); n = 1/7	0.3 ± 0 (0 %); n = 0/6	5 ± 8.2 (33.3 %); n = 2/6
Vancomycin	32.2 ± 39 (40 %); n = 2/5	3 ± 3.2 (60 %); n = 3/5	26.9 ± 37.5 (33.3 %); n = 1/3	25.3 ± 25 (50 %); n = 1/2

Values represent mean ± standard deviation (proportion of patients with a detectable toxin concentration)

Overall, a decrease in both toxin A and B were observed for patients given both fidaxomicin and vancomycin by days 3–5 (midpoint of therapy). Toxin A concentrations remained low for patients given fidaxomicin, but not vancomycin through the follow-up period including two patients who developed CDI recurrence (subjects 5 and 12; Fig. 2a, b). Toxin B concentrations increased at follow-up in patients with CDI recurrence in both fidaxomicin- (subjects 5 and 12) and vancomycin-treated (subject 3) groups (Fig. 3a, b).

### Toxin A and B concentrations and clinical outcomes: clinical cure

Toxins concentrations were compared between the two groups with respect to clinical outcomes at end of therapy and follow-up (Table 3). Within the group of patients who were cured by the end of therapy, both fidaxomicin and vancomycin were associated with low concentrations of toxin B at midpoint versus the failed patients (subjects 2 and 10) while toxin A concentrations were low at midpoint for all patients regardless of end of therapy outcome.

**Table 3:** *C. difficile* toxin A and B concentrations based on clinical outcomes

Period	Outcome measure	Therapy	Baseline	Mid-point	End-of-therapy	Follow-up
End of therapy	Cured	Fidaxomicin	A: 40.2 ± 39.8 (50 %); n = 3/6 B: 48.8 ± 28.8 (100 %); 6/6	A: 0.7 ± 1 (0.2 %); n = 1/6 B: 5.2 ± 10.8 (0.2 %); n = 1/6	A: 0.3 ± 0 (0 %); 0/6 B: 0.3 ± 0 (0 %); 0/6	N/A
		Vancomycin	A: 28.2 ± 36.6 (100 %); n = 3/3 B: 26.9 ± 37.6 (33.3 %); n = 1/3	A: 0.3 ± 0 (0 %); n = 0/3 B: 0.8 ± 0.7 (33.3 %); n = 1/3	A: 19.1 ± 26.6 (33.3 %); n = 1/3 B: 26.9 ± 37.6 (33.3 %); n = 1/3	N/A
	Failed	Fidaxomicin^a^	N/A	N/A	N/A	N/A
		Vancomycin	A: 25.5 ± 21.5 (100 %); n = 2/2 B: 40.2 ± 56.3 (50 %); n = 1/2	A: 0.3 ± 0 (0 %); n = 0/2 B: 6.4 ± 3.3 (100 %); n = 2/2	N/A	N/A
Follow-up	Sustained cure	Fidaxomicin	A: 20.2 ± 34.5 (25 %); n = 1/4 B: 33.3 ± 22.9 (100 %); n = 4/4	A: 0.3 ± 0 (0 %); n = 0/4 B: 0.3 ± 0 (0 %); n = 0/4	A: 0.3 ± 0 (0 %); n = 0/4 B: 0.3 ± 0 (0 %); n = 0/4	A: 0.3 ± 0 (0 %); n = 0/4 B: 0.3 ± 0 (0 %); n = 0/4
		Vancomycin^b^	A: 2.2 (100 %); n = 1/1 B: 0.3 (0 %); n = 0/1	A: 0.3 (0 %); n = 0/1 B: 0.3 (0 %); n = 0/1	A: 0.3 (0 %); n = 0/1 B: 0.3 (0 %); n = 0/1	A: 0.3 (0 %); n = 0/1 B: 0.3 (0 %); n = 0/1
	CDI recurrence	Fidaxomicin	A: 80 ± 0 (100 %); n = 2/2 B: 80 ± 0 (100 %); n = 2/2	A: 1.6 ± 1.3 (50 %); n = 1/2 B: 15 ± 14.5 (50 %); n = 1/2	A: 0.3 ± 0 (0 %); n = 0/2 B: 0.3 ± 0 (0 %); n = 0/2	A: 0.3 ± 0 (0 %); n = 0/2 B: 14.2 ± 8.5 (100 %); n = 2/2
		Vancomycin^b^	A: 2.5 (100 %); n = 1/1 B: 0.3 (0 %); n = 0/1	A: 0.3 (0 %); n = 0/1 B: 1.7 (100 %); n = 1/1	A: 56.7 (100 %); n = 1/1 B: 80 (100 %); n = 1/1	A: 70.8 (100 %); n = 1/1 B: 50.4 (100 %); n = 1/1

Values represent mean ± standard deviation (proportion of patients with a detectable toxin concentration)

The lower and upper limits of detection were 0.3 and 80 ng/mL, respectively, for both toxin A and B

*CDI* *Clostridium difficile* infection, *N/A* not applicable

^a^None of fidaxomicin patients failed therapy at the end of the 10-day treatment course

^b^In the vancomycin group, one patient experienced sustained cure while one developed CDI recurrence, thus standard deviation is not reported

### CDI recurrence

Low concentrations of toxins A and B were observed in the fidaxomicin group patients who remained in remission at follow-up and those who relapsed compared with the concentrations observed in the patient who relapsed in the vancomycin group (Table 2). This difference primarily arose from the reduction in toxins concentrations in the relapsed patients, where the two patients who initially received fidaxomicin had very low concentrations of toxin A (subjects 5 and 12), while the patient who previously received vancomycin had a concentration of 70.8 ng/mL (subject 3). Mean toxin B concentration in the fidaxomicin patients who experienced CDI recurrence was lower than the concentration measured in the follow-up sample from the patient who was initiated on vancomycin and later relapsed. Of note, the follow up stool sample of subject 5 was collected within 3 days of initiating the treatment for the recurrent infection, while that of subject 12 was collected prior to the initiation of therapy. Both patients were treated with a combination therapy of vancomycin and metronidazole. The follow up stool sample of subject 3 was collected within 24 h of initiating therapy with vancomycin. Both colonized and non-colonized patients who remained in remission at follow-up in the fidaxomicin group (subjects 29 and 33) did not have detectable concentrations of either toxin in their stools.

### Correlation of stool and microbiologic characteristics with *C. difficile* toxin concentrations

When the correlation between faecal bacterial load of *C. difficile* was studied with toxins A and B concentrations at all time points combined, significant positive correlation was observed with both toxins in the presence of vegetative cells (*r*_*s*_ = 0.5, *P* = 0.003, for toxin A and *r*_*s*_ = 0.3, *P* = 0.007, for toxin B). Moreover, toxin B, but not toxin A, was significantly associated with more loose stools (*r*_*s*_ = 0.5, *P* < 0.001, versus *r*_*s*_ = 0.3, *P* = 0.07). Additionally, toxin B was weakly associated with more frequent bowel movements (*r*_*s*_ = 0.3, *P* = 0.05) while toxin A did not demonstrate such correlation (*r*_*s*_ = 0.2, *P* = 0.2).

## Discussion

While antibiotics with activity against *C. difficile* are the mainstay of therapy for CDI, agents with additional anti-toxin effects may provide an added benefit in the management of the disease. In an attempt to assess the effect of conventional therapies on the dynamic process of toxins production in vitro, Babakhani and colleagues utilized sub-MIC concentrations of fidaxomicin, vancomycin, and metronidazole to compare *C. difficile* toxin production without affecting the bacterial growth [6]. Fidaxomicin was the only agent to inhibit the expression of the genes (*TcdA* and *TcdB*) that code for the production of toxin A and toxin B presumably via its macrolide based mechanism of transcription inhibition. This finding may explain the observations seen in the current study in which fidaxomicin was associated with substantial overall reduction of both toxins as opposed to vancomycin in patients with CDI. The effect of reducing toxin B concentrations by fidaxomicin was also observed by Louie et al. [12]. In contrast to their study, which only assessed toxin B concentration, we assessed the change in both toxins A and B concentrations over time from the start of the CDI episode until after 30 days post initiation of anti-CDI treatment. We found that fidaxomicin was associated with sustained reduction of both *C. difficile* toxins over the course of the disease until the follow-up even in the two patients who developed CDI recurrence where they maintained undetectable concentrations of toxin A and low concentrations of toxin B.

The positive correlation of *C. difficile* bacterial load with toxins concentrations observed in the current study is consistent with a finding by Åkerlund et al. [5]. This observation helps elucidating the minor effect of vancomycin on reducing toxins concentrations which is presumed to occur through bacterial killing, whereas fidaxomicin acts on both the bacteria and the toxin production mechanism. Additionally, similar to the finding by Åkerlund et al. we found a significant correlation between toxin B concentrations and frequent bowel movements aiding the general notion that *C. difficile* toxins are the major drive of the disease severity.

Although our study included a limited number of patients, the use of our unique stool sampling scheme combined with toxin concentration determination provides some novel insight. Notably, we showed that fidaxomicin was associated with reduction in the overall concentrations of *C. difficile* toxins up to more than 30 days post therapy initiation regardless of the clinical outcome as compared with vancomycin. Additionally, the elimination of *C. difficile* toxins may also aid in relieving the diarrheal symptoms of the disease since their concentrations were highly correlated with stool consistency and frequency. For example, the two patients who had CDI recurrence who initially received fidaxomicin had less frequent and more formed stools compared with their counterpart patient who initially treated with vancomycin. The study was also limited by the number of comparators as looking at the effect of other anti-CDI agents, such as metronidazole and rifaximin, may provide additional understanding of their effect on *C. difficile* toxins concentrations. Moreover, the current study only enrolled patients admitted to the hospital (i.e., patients presenting with severe form of the disease). Therefore, conducting a similar study on outpatients may elaborate these outcomes in a CDI population who are less sick and presenting with mild-to-moderate CDI. Based on the results of this study, we conclude that fidaxomicin can be considered a viable anti-CDI agent for patients experiencing severe form of the disease since it was associated with lower concentrations of both *C. difficile* toxins. A study done on a larger patient population can help further exploration of this outcome.
